# Fatty change and muscle atrophy in patients with rotator cuff tears: a prospective study with a mean 6-year follow-up

**DOI:** 10.1016/j.jseint.2026.101624

**Published:** 2026-01-12

**Authors:** Atsushi Arino, Nobuyuki Yamamoto, Jun Kawakami, Rei Kimura, Hideaki Nagamoto, Hirotaka Sano, Toshimi Aizawa, Eiji Itoi

**Affiliations:** a Department of Orthopaedic Surgery, Tohoku University School of Medicine, Sendai, Miyagi, Japan; b Department of Sports Medicine and Reconstruction of Motion Function, Tohoku University School of Medicine, Sendai, Miyagi, Japan; c Graduate School of Sports Science, Waseda University, Tokorozawa, Saitama, Japan; d Department of Orthopaedic Surgery, Sendai City Hospital, Sendai, Miyagi, Japan; e Department of Orthopaedic Surgery, Tohoku Rosai Hospital, Sendai, Miyagi, Japan

**Keywords:** Rotator cuff tear, Fatty change, Muscle atrophy, Occupation ratio, Supraspinatus, Magnetic resonance imaging

## Abstract

**Background:**

Understanding how fatty change and muscle atrophy progress—and how they are interrelated—is important for determining appropriate treatment strategies in patients with rotator cuff tears. To prospectively evaluate the relationship between supraspinatus muscle atrophy and fatty change in patients with symptomatic rotator cuff tears treated nonoperatively, using magnetic resonance imaging.

**Methods:**

Among 225 patients with symptomatic rotator cuff tears treated nonoperatively between 2006 and 2015, 58 patients (59 shoulders) who underwent at least two magnetic resonance imaging evaluations and were followed for ≥30 months were included. The occupation ratio (cross-sectional area of supraspinatus/supraspinatus fossa) was calculated to assess muscle atrophy, and fatty change was graded using the Goutallier classification. Tear dimensions (length and width) were also measured.

**Results:**

Muscle atrophy progressed in 40 shoulders (68%), and fatty change progressed in 7 shoulders (12%) during an average duration of 53 months. Fatty change progression was significantly associated with larger initial tear length (25.9 mm vs. 11.0 mm; *P* < .001). The occupation ratio decreased progressively with higher Goutallier grades (*P* = .020).

**Conclusion:**

Fatty change progressed in 7 shoulders (12%) during an average duration of 53 months. It was strongly associated with larger baseline tear size, tear enlargement, and muscle atrophy progression, highlighting the importance of early identification.

Rotator cuff tears are common in individuals over 50 years of age, with prevalence increasing with age. The prevalence has been reported to be 10.7% in individuals in their 50s and 36.6% in those in their 80s.^17^^,^^24^ Nonoperative treatment is often selected for patients with atraumatic rotator cuff tears. Clinical studies have demonstrated that conservative management—including injections, medications, and rehabilitation—is effective in over 70% of patients.^5^^,^^8^^,^^11^^,^^13^

Despite recent advances in understanding the natural history of degenerative rotator cuff tears, risk factors for tear progression and pain development remain unclear. Understanding this natural history is important for selecting optimal treatment strategies. While the prevalence of rotator cuff tears in the general population is well documented, controversy persists regarding the management of symptomatic cases.^4^ Both nonsurgical and surgical treatment strategies have evolved significantly over time.^21^

It is well established that large, chronic rotator cuff tears are more likely to result in significant fatty change in the rotator cuff muscles than small tears. However, the timeline of these degenerative changes and the associated risk factors remains to be clarified. Fatty changes are clinically significant because they are associated with inferior clinical outcomes and lower tendon-healing rates after surgical repair.^2^^,^^7^^,^^19^ Some authors have suggested that surgical repair should be performed before the development of advanced fatty change, particularly beyond Goutallier grade 2.^16^

To comprehensively understand the progression of fatty change, long-term observation of the natural history of rotator cuff tears is necessary. Although several studies have investigated this issue,^5^^,^^10^^,^^15^^,^^18^^,^^20^^,^^23^ only two have used magnetic resonance imaging (MRI) to directly evaluate the progression of fatty change.^5^^,^^15^ However, both were limited by small sample sizes and relatively short follow-up durations.

The purpose of this study was to prospectively investigate the relationship between muscle atrophy and fatty change in patients with symptomatic rotator cuff tears using MRI. We hypothesized that (1) there would be a correlation between fatty change and muscle atrophy, and (2) patients with progressive fatty change would exhibit greater muscle atrophy and a more rapid rate of atrophic progression.

## Methods

### Subjects

Between January 2006 and October 2015, a total of 225 consecutive patients with symptomatic rotator cuff tears were referred to our institution. Of these, 58 patients (59 shoulders) who met the following inclusion criteria were prospectively enrolled: (1) presence of a painful rotator cuff tear, either full-thickness or partial-thickness, at the initial visit; (2) availability of at least two MRI examinations; (3) a minimum follow-up period of 30 months; and (4) treatment by nonoperative means.

Exclusion criteria were: (1) prior shoulder surgery; (2) absence of shoulder pain at the initial visit; and (3) symptoms attributable to conditions other than rotator cuff tears, such as cervical spine pathology, glenohumeral arthritis, or inflammatory arthritis. All patients received nonoperative treatment, including oral medications, corticosteroid injections into the subacromial bursa or glenohumeral joint, and physical therapy. Patients with traumatic injuries or those under the age of 50 were excluded from conservative treatment. All included patients were advised to attend regular follow-up visits at our institution, ideally every six months, regardless of symptom improvement. Surgical intervention was considered if no improvement was observed after at least three months of nonoperative care. In practice, most exclusions from the analytic cohort reflected insufficient follow-up duration (short follow-up) rather than clinical ineligibility.

MRI scans were scheduled every six months during the first two years and annually thereafter. This study was approved by the institutional review board of our hospital (approval number: #2013-1-140). At the time of the initial MRI, the mean patient age was 64 years (range, 50-80 years). The cohort included 29 male and 30 female patients. There were 21 full-thickness tears and 37 partial-thickness tears, further classified into 17 bursal-sided, 14 articular-sided, and 7 intratendinous tears. The mean follow-up duration was 72 months (range, 30-147 months). Baseline demographics and initial MRI findings are summarized in Table I. All rotator cuff tears were confirmed on MRI.

**Table I:** Patient demographics and baseline MRI findings.

Variable	Value
Number of shoulders	59
Age (yr)	64.0 ± 8.4
Sex	
Male	29
Female	30
Follow-up (mo)	72.0 ± 27.8
Tear size at first MRI	
Length (mm)	12.8 ± 10.8
Width (mm)	9.5 ± 8.3
Tear type	
Full-thickness	21 (36%)
Partial-thickness	
Bursal side	17 (29%)
Articular side	14 (24%)
Intratendinous	7 (12%)
Change of tear size	
Length (mm)	6.0 ± 7.0
Width (mm)	2.8 ± 5.5
Supraspinatus fatty change at first MRI (Goutallier classification^6^)	
Grade 0	18 (31%)
Grade 1	35 (59%)
Grade 2	3 (5%)
Grade 3	1 (2%)
Grade 4	2 (3%)
Supraspinatus muscle atrophy at first MRI (%)	58.8 ± 12.3

*MRI*, magnetic resonance imaging.

Values are presented as mean ± standard deviation unless otherwise indicated.

### Magnetic resonance imaging evaluation

All patients underwent shoulder MRI using a 3.0-tesla imaging system (Intera Achieva 3.0T; Philips Medical Systems, Amsterdam, Netherlands). The imaging protocol included fat-saturated T2-weighted turbo spin echo images (4.0-mm slice thickness, 0.35-mm interslice gap, 140-mm field of view, 448 × 324 matrix). All MRI evaluations were performed in a blinded fashion by an experienced orthopedic surgeon. Tear size was measured in the sagittal and coronal planes using standardized image scaling tools. For partial-thickness tears, measurements were taken in all three orthogonal planes, whereas coronal and sagittal images were used for full-thickness tears. Tear length was defined as the maximum mediolateral (ML) extent, and width was defined as the maximum anteroposterior (AP) extent. Tear size was categorized according to the Cofield classification: small (<1 cm), medium (1-3 cm), large (3-5 cm), and massive (>5 cm).^3^

Muscle atrophy was assessed by measuring the cross-sectional area of the supraspinatus muscle using ImageJ software (National Institutes of Health, Bethesda, MD, USA). The occupation ratio was calculated as the percentage of supraspinatus area occupying the supraspinatus fossa. Fatty change of the supraspinatus was graded according to the Goutallier classification.^6^

### Statistical analysis

Upon examination of the initial MRI results comparing fatty change and muscle atrophy of the supraspinatus muscle, variables were analyzed using the Kruskal–Wallis test and Bonferroni post hoc test. Boxplots and scatter plots were employed to illustrate the statistical distributions. A comparison of baseline data between the fatty change progression and nonprogression groups was conducted using the chi-square test for categorical variables and the Wilcoxson singed-rank test for normally distributed continuous variables. All analyses were performed using the R software version 4.2.0. Statistical significance was set as *P* < .05. In addition, AP (“width”) and ML (“length”) tear enlargement rates (mm/year) were computed as Δ (mm) divided by follow-up duration in years; because fatty-change onset is interval censored between MRI time points, these rates are reported as complementary dynamic measures rather than exact onset times.

## Results

**Figure 1:**
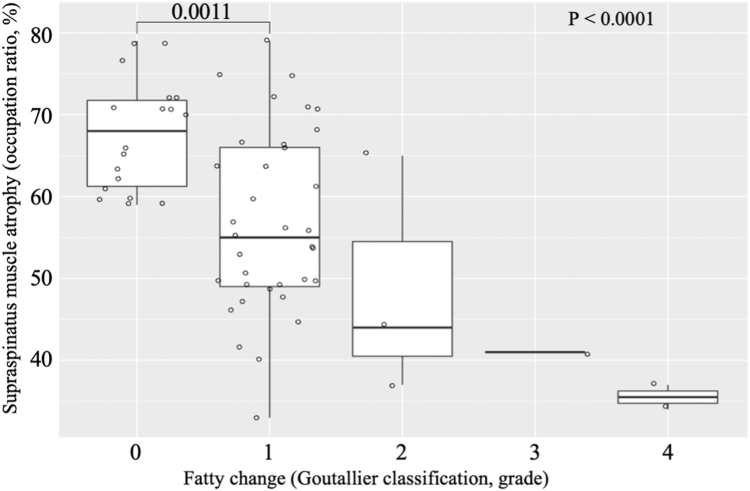
Relationship between fatty change and muscle atrophy of supraspinatus at first MRI. Standard box plot with a scatterplot of the supraspinatus fatty change (Goutallier classification^6^) and supraspinatus muscle atrophy (occupation ratio, %). The box plots span the interquartile range. The horizontal line inside the box represents the mean. *MRI*, magnetic resonance imaging.

Muscle atrophy showed a significant relationship with the degree of fatty change based on the Goutallier classification (*P* = .008). The mean occupation ratio of the supraspinatus muscle was 65.7 ± 0.6% in grade 0, 56.3 ± 1.1% in grade 1, 43.4 ± 1.0% in grade 2, 29.4 ± 9.1% in grade 3, and 33.3 ± 0.3% in grade 4 (Fig. 1). Bonferroni post hoc analysis revealed a significant difference between grades 0 and 1 (*P* = .0011).

Patients were then divided into two groups: those with fatty change progression and those without. There were no significant differences between the groups in terms of age, sex, follow-up duration, or tear type. However, significant differences were found in tear size at the initial MRI, both length (*P* < .001) and width (*P* = .001), as well as in tear size progression—length (*P* = .006) and width (*P* = .028). Fatty change grade at the initial MRI (*P* = .020) and progression of supraspinatus muscle atrophy (*P* = .0011) also differed significantly between the two groups. In addition, patients in the fatty change progression group were more likely to have medium or large tears at baseline (*P* = .025). Both tear size and muscle atrophy showed significant progression between the initial and final MRI in this group (Table II). Time-normalized tear enlargement rates were higher in progressors vs. nonprogressors: AP 2.18 vs. 1.17 mm/year and ML 1.23 vs. 0.48 mm/year, respectively.

**Table II:** Comparison of baseline and follow-up variables between the fatty change progression and nonprogression groups.

Variable	Fatty change progression		*P* value
	(+)	(−)	
Number of shoulders	7	52	
Mean age (yr)	66.3 ± 10.8	63.7 ± 8.1	.440
Sex = male	6 (86%)	23 (44%)	.097
Follow-up (mo)	68.0 ± 12.7	72.6 ± 29.3	.683
Tear size at first MRI			
Length (mm)	25.9 ± 13.7	11.0 ± 9.2	<.001
Width (mm)	18.6 ± 12.2	8.3 ± 6.9	.001
Tear type			.128
Full-thickness	5 (71%)	16 (31%)	
Partial-thickness			
Bursal side	2 (29%)	15 (29%)	
Articular side	0 (0%)	14 (27%)	
Intratendinous	0 (0%)	7 (14%)	
Progression of tear size			
Length (mm)	12.6 ± 6.9	5.1 ± 6.5	.006
Width (mm)	7.1 ± 7.6	2.3 ± 5.0	.028
Supraspinatus Goutallier grade			.020
Grade 0	0 (0%)	18 (35%)	
Grade 1	5 (71%)	30 (58%)	
Grade 2	1 (14%)	2 (4%)	
Grade 3	1 (14%)	0 (0%)	
Grade 4	0 (0%)	2 (4%)	
Supraspinatus muscle atrophy at first MRI (%)	47.4 ± 5.9	60.3 ± 12.1	.008
Progression of supraspinatus muscle atrophy (%)	14.7 ± 8.4	3.5 ± 10.7	.011

*MRI*, magnetic resonance imaging.

Values are presented as mean ± standard deviation unless otherwise indicated.

Overall, muscle atrophy progressed in 40 of 59 shoulders (68%). Fatty change progression was observed in 7 shoulders (12%). Of these, 5 shoulders exhibited a one-grade increase in Goutallier classification (three shoulders progressed from grade 1 to 2, 1 from grade 2 to 3, and 1 from grade 3 to 4), and 2 shoulders showed a two-grade progression (from grade 1 to 3). The average time to observe fatty change progression was 53 months (range, 50-87 months). Among the 7 shoulders with fatty change progression, 5 had full-thickness tears and 2 had bursal-side partial-thickness tears at the initial MRI. Fatty change progression therefore occurred in 5/21 full-thickness tears (24%) and 2/38 partial-thickness tears (5.3%) (Tables II and III). The distribution of supraspinatus Goutallier grades at the first and final MRI is summarized in Table IV. Fatty change progression occurred in 0/18 grade 0 shoulders, 5/35 grade 1 shoulders, 1/3 grade 2 shoulders, 1/1 grade 3 shoulders, and 0/2 grade 4 shoulders at baseline, indicating that progression predominantly arose from shoulders with mild to moderate fatty change.

**Table III:** Duration and pattern of fatty change progression in individual cases.

Variable	Case #1	Case #2	Case #3	Case #4	Case #5	Case #6	Case #7
Tear type	Full	Articular	Full	Full	Full	Full	Full
Goutallier classification							
First MRI	1	1	1	1	1	2	3
Final MRI	2	2	2	3	3	3	4
Muscle atrophy (occupation ratio, %)							
First MRI	40	54	54	50	48	43	40
Final MRI	28	39	45	33	22	20	39
Duration (mo)							
Total	57	63	76	68	75	87	50
Grade 1 → 2	57	63	76	25	58		
Grade 2 → 3				43	17	87	
Grade 3 → 4							50

*MRI*, magnetic resonance imaging.

**Table IV:** Breakdown of changes in fatty change.

Supraspinatus Goutallier grade	First MRI	Final MRI
Grade 0	18	18
Grade 1	35	30
Grade 2	3	5
Grade 3	1	3
Grade 4	2	3

*MRI*, magnetic resonance imaging.

## Discussion

This prospective study investigated the natural course of fatty change in patients with symptomatic rotator cuff tears treated nonoperatively over an average follow-up period of six years. Our findings revealed three key observations: (1) progression of fatty change was significantly associated with supraspinatus muscle atrophy; (2) patients who exhibited fatty change progression had significantly larger initial tear sizes and more pronounced atrophic changes; and (3) the average time to detect Goutallier grade progression was 53 months. Fatty change progression was observed in 12% of shoulders, with one-grade changes in five cases and two-grade changes in two cases. Because most exclusions were due to short follow-up, any selection bias likely attenuates observed long-term progression (bias toward the null). Given interval-censored imaging schedules, we interpret fatty change timing cautiously and provide mm/year tear enlargement rates as a pragmatic dynamic indicator. For clinical practice, risk-stratified surveillance is suggested: large or full-thickness tears and early atrophic changes merit closer MRI follow-up at 6- to 12-month intervals initially, whereas small partial-thickness tears without adverse muscle quality may be monitored less frequently.

Prior studies have documented a correlation between muscle atrophy and fatty change.^1^^,^^7^^,^^12^^,^^14^ Our results similarly demonstrated that advanced muscle atrophy was more likely to accompany fatty change progression. Notably, the Bonferroni post hoc analysis indicated a statistically significant difference only between Goutallier grades 0 and 1. This finding may reflect the high proportion of patients clustered in lower Goutallier grades at baseline, with relatively few exhibiting grades ≥2.

Two previous studies using MRI have examined the progression of fatty change in rotator cuff tears. Maman et al^15^ followed 49 shoulders for 20 months and reported a 20% rate of progression, while Fucentese et al^5^ monitored 24 shoulders for 42 months and found a 25% progression rate. In contrast, our study followed 59 shoulders for a substantially longer period of six years and found a lower progression rate of 12%. This difference may be partly attributable to the higher prevalence of partial-thickness and small tears in our cohort (64%), compared to 44% and 0% in the Maman and Fucentese studies, respectively. However, due to differences in study design, follow-up duration, and patient demographics, direct comparisons should be interpreted with caution.

The clinical relevance of our findings lies in the question of how and when patients with conservatively managed rotator cuff tears should be monitored for structural progression. Yamaguchi et al^22^ recommended follow-up ultrasonography every six months, then annually, for asymptomatic tears. However, given the relatively low rate and slow progression of fatty change in our study, such frequent monitoring may not be necessary in all cases. A more individualized follow-up protocol based on baseline tear size, type, and muscle quality may be more appropriate and cost-effective.

Our study has several strengths, including a prospective design, relatively large sample size, long follow-up duration, and the use of high-resolution MRI, which is highly accurate for detecting both full- and partial-thickness tears.^9^ Although some studies have used ultrasonography to monitor tear progression,^7^ this modality is less reliable for detecting changes in partial-thickness or articular-sided tears.

Nevertheless, this study has limitations. First, the patient cohort had a higher proportion of partial and small tears compared with prior studies,^5^^,^^15^ which may limit generalizability. Second, the Goutallier classification is subjective and prone to interobserver variability. Its categorical scale may lack sensitivity to subtle changes. More quantitative and objective methods are needed for accurate assessment. Third, the pattern and timing of tear progression between MRI intervals remain unclear. Shorter-interval imaging could provide more granular data but may be impractical due to cost and patient burden. In this context, ultrasonography may serve as a useful adjunct, particularly for tracking full-thickness tears. Additional limitations include the absence of multireader/replicate reliability testing in this version and incomplete clinical outcome scales; both are planned for subsequent work.

## Conclusion

During an average follow-up of six years, fatty change progressed in 7 of 59 shoulders (12%), with a mean time to progression of 53 months. Fatty change was associated with muscle atrophy, and patients with progressive fatty change demonstrated significantly larger initial tears, greater tear progression, and more severe muscle atrophy than those without progression.

## Disclaimers

Funding: No funding was disclosed by the authors.

Conflicts of interest: The authors, their immediate families, and any research foundation with which they are affiliated have not received any financial payments or other benefits from any commercial entity related to the subject of this article.
